# Pregnant Turkish Muslim Women’s Perspectives and Experiences on Religion and Spirituality during Pregnancy

**DOI:** 10.1007/s10943-025-02258-x

**Published:** 2025-01-31

**Authors:** Figen Alp Yilmaz, Tuğba Uzunçakmak

**Affiliations:** 1 Faculty of Health Sciences, Nursing Department Alanya, Alanya Alaaddin Keykubat University, Alanya, Turkey; 2 Faculty of Health Sciences, Nursing Department Yozgat, Yozgat Bozok University, Yozgat, Turkey

**Keywords:** Pregnancy, Religion, Spirituality

## Abstract

The aim of this study is to determine the views and experiences of pregnant Muslim women about religion and spirituality during pregnancy. A descriptive, phenomenological design was used, and the study was conducted with 16 pregnant Muslim women living in Türkiye. The thematic analysis method was used. The main themes created in the study were pregnant women’s motivation to have children, changes during pregnancy, faith and spirituality during pregnancy, support for the moment of birth, and motherhood. Pregnant women also need moral support in addition to routine practices and follow-ups during this difficult and distressing process. Religious practices provide psychological comfort for pregnant women in coping with the symptoms they experience and having a healthy birth and a healthy baby. Spiritual care should be given importance in raising health professionals and spirituality-based topics should be included in the education process.

## Introduction

Pregnancy is a complex and special process that involves many physiological, biological, psychological, and social changes. Pregnant women must adapt to these changes to go through this process healthily (Monterrosa et al., 2023). They use relaxation techniques, such as support from family and friends, psychological support, and meditation, to adapt to this new life and cope with the stressors that arise during this period (Mutmainnah & Afiyanti, 2019). In countries where religion is dominant, the leading sources of coping with life changes are religious and spiritual orientations (Başar, 2022).

Türkiye is a country with a large Muslim population and believers in Islam. The religion of Islam aims to bring the person closer to God, as well as developing his/her values and self-realization. Having a conscious and constant relationship with God means living daily life according to the rules set by God in the holy book of Islam Quran and Hadiths (Mutmainnah & Afiyanti, 2019). Having spirituality in Islam also means being as good a person as God desires. Spirituality is therefore defined as something that has an individual, motivational, and relational ‘face’. Relationship with God is the main theme of spirituality in Islam. The nature of spiritual relationships consists of beliefs, worship, and morality/daily behavior. Spirituality also encompasses the concept of self, the meaning of life, and relationships with others and nature (Babanazari, 2017; Cyphers et al., 2016). Due to the miraculous nature of the pregnancy process, the increase in women’s spiritual commitment during this period causes the role of spirituality to deepen (Bawadi & Al-Hamdan, 2017; Ohaja et al., 2019). In this context, pregnant women who adhere to religion, hoping to receive divine protection from a higher power through religious practices and prayers, have a healthier pregnancy (Crowther et al., 2015; Aziato et al., 2018). At the same time, it is reported that spiritual practices performed during pregnancy connect the mother to life and reduce the negative effects of the changes that occur during the pregnancy on the health of the mother and the fetus (Başar, 2022).

The pregnant woman’s spiritual practices and religious beliefs during this period make it easier to cope with stress and are important to her as they will reduce the negativities that may be experienced (Aziato et al., 2016). Understanding experiences is difficult because it requires having an idea about people’s perceptions, reactions, and the effects of the social environment (Bandura, 1986). Therefore, it is important to conduct a qualitative study to examine the views and experiences of pregnant Muslim women about religion and spirituality during pregnancy. This qualitative study was conducted to determine the views and experiences of pregnant Muslim women in Türkiye about religion and spirituality during pregnancy.

## Methods

### Study Design

Since the phenomenological approach focuses on people’s experiences with a phenomenon, this qualitative study is based on this approach. Phenomenological studies are often conducted to shed light on health issues. The phenomenon examined in this study is pregnant women’s views on the impact of religion and spirituality during pregnancy. The study was reported in accordance with the Standards for Reporting Qualitative Research.

### Recruitment and Sampling

The study was carried out between October and December 2023. The sample of the study consisted of pregnant women who presented to the gynecology clinic of a university hospital in the Central Anatolia Region. The purposive sampling method was used to select women. This method allows the selection of information-rich situations in line with the purpose of the study to conduct in-depth research. In qualitative research, sample size is determined by continuing to collect data until the concepts start to appear repeatedly (Houser, 2016). The number of participants in this study was determined according to the "data saturation" principle, which is commonly employed in qualitative research. The study included pregnant women who were over 18 years of age, could speak and understand Turkish, did not have any psychiatric disorders, had a healthy pregnancy, and did not have any chronic diseases. The sample included 16 pregnant women who met these conditions. Exclusion criteria were having a high-risk pregnancy, having a chronic disease, and refusing to participate in the study.

### Data Collection

The data of the research were collected using the semi-structured in-depth interview method, which is the most commonly utilized in qualitative research. Data collection tools were a descriptive information form and a semi-structured questionnaire. The descriptive information form included questions about sociodemographic characteristics such as age and income. During the development of the forms, categories were determined by examining other studies on the subject and problem areas, and eight open-ended questions covering these headings were created. A pilot study was conducted with five pregnant women to review the interview process and questions, and these interviews were not used in the analysis. The interviews were held by a female researcher experienced in qualitative research. They were carried out face to face in a quiet room so that the pregnant women could express themselves more easily and without distraction. At the beginning of the interviews, the participants were informed about the purpose, scope, ethical aspects, and potential benefits of the study. To clarify participants’ answers, increase confirmability, and give participants a chance to change their answers, the researcher asked them to read and confirm a summary of their responses after each question. Each interview lasted 30–40 min and was audio recorded.

### Data Analysis

Data regarding sociodemographic characteristics were expressed in numbers and percentages. All data collected through the semi-structured form were analyzed using a thematic analysis. The interview recordings were transcribed verbatim. Data analysis was carried out separately by two researchers. Coding the data is an important stage of the thematic analysis. It helps classify data and improve connections between researchers (Cresswell & Poth, 2018). In this research, Clark and Braun’s thematic analysis stages were employed for data analysis. This analysis method consists of six stages, which are (1) becoming familiar with the data, (2) creating initial codes, (3) eliciting themes, (4) reviewing the themes, (5) defining and naming the themes, and (6) preparing the report (Braun & Clarke, 2014).

### Rigor and Trustworthiness

The study was reported according to the Standards for Reporting Qualitative Research (SRQR) (O’Brien et al., 2014). In qualitative research, the credibility of the results is considered one of the most important criteria of scientific research. "Validity," "reliability," "credibility," and "confirmability" are the most commonly used criteria in research in this respect. In this research, the criteria suggested by Coloraf and Evans (2016) were taken into account for validity and reliability. The two researchers had doctoral degrees, had attended qualitative research courses, and were experienced in qualitative research. To ensure reliability, all interviews were conducted by the same researcher. The interviews were held in a quiet environment where the woman and the interviewer were alone. During the interviews, enough time was allocated to each participant to express their feelings and thoughts comfortably. To ensure validity, all interview records were coded by the two researchers without adding any comments, following reliability criteria. The originality of the data was preserved and the data were reported comprehensively by presenting participants’ direct statements.

### Ethical Considerations

The permission of the social sciences ethics committee of the Yozgat Bozok University (Number:38/10) where the research was conducted was obtained. In addition, the principle of voluntariness in participating in the research was taken into account, and verbal and written consent of the individuals included in the research were obtained.

##  Results

According to the findings, 68.8% of the pregnant women in the study were between the ages of 23–30, 43.8% were university graduates, 75% described their income as equal to their expenses, and 75% were housewives. The duration of marriage was 2–9 years in 75%, 75% of them had 1–2 pregnancies, 50% were in the 11th to 29th weeks of pregnancy, and 75% stated that their pregnancy was planned (Table 1).

**Table 1:** Sociodemographic characteristics of the pregnant women (*n* = 16)

Characteristics	*N*	%
Age groups (years)		
23–30	11	68.8
31–36	5	31.3
Education level		
Primary school	1	6.3
Secondary school	3	18.8
High school	5	31.3
University	7	43.8
Income		
Income < expenses	1	6.3
Income = to expenses	12	75.0
Income > expenses	3	18.8
Job		
Housewife	12	75.0
Civil servant	4	25.0
Duration of marriage (years)		
2–9	12	75.0
10–20	4	25.0
Number of pregnancies		
1–2	12	75.0
3–4	4	25.0
Gestational age (week)		
11–29	8	50.0
30–38	8	50.0
Status of the pregnancy		
Planned	12	75.0
Unplanned	4	25.0

**Fig. 1:**
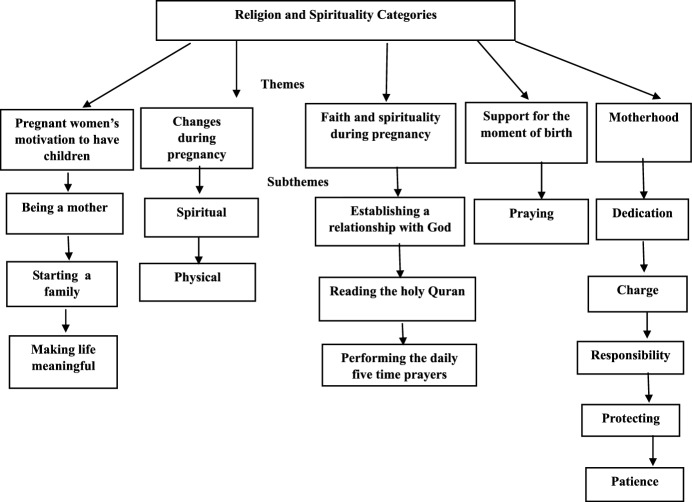
Religion and spirituality themes

The category themes and sub-themes related to the religion and spirituality dimension of pregnant women are shown in Fig. 1.

## Pregnant Women’s Motivation to have Children

### Being a Mother

*Frankly, not everyone has the opportunity to give birth to a child and raise an individual. As God has bestowed it upon us as a woman, I have wanted to experience it once more (P2).*

*When I got married, I did not have such a thought at all and thought it would not happen. I don’t know whether it is in the essence of a woman or her structure, but as I saw people getting married and having children around me, I started to develop feelings of motherhood (P3).*

### Starting a Family

*My husband and I decided to have children. We thought we could only truly be a family when we had children. We love children very much. It is very nice to have a big family. If God pleases, we want two or three children (P4).*

### Making Life Meaningful

*When individuals want to give something to the world or when they want to add something to life, they think that they can do this by having a child, and I believe that by bringing a child into the world, I will be a more useful person to the world, which is one of the goals of marriage. I was grateful when I heard that I was pregnant (P5).*

## Changes During Pregnancy

### Spiritual

*When you contemplate spiritually, you feel a living thing inside, coming from a drop of water. It is growing; you feel the heartbeats. There is something that has not existed before, with the wisdom of God. You start contemplating deeply. You think about the divine wisdom; I mean what a great thing it is (P1).*

*Spiritual change really happens. For example, I see very beautiful dreams. We also know that pregnant women are protected. As soon as a woman becomes pregnant, an angel appears on the mother’s shoulders to protect her (P2).*

*I wasn’t this emotional before; now I’ve become much more emotional. I get sensitive at the slightest thing and start weeping. I’ve become very touchy (P7).*

*My mood is not stable. I’m either very irritated or very emotional; it is tiring (P5).*

*Our elderly in the family of my husband and mine, neighbors, and friends are all very supportive. They share their experiences about what to eat, what to do or not to do. I really like this support. It is reducing my fears and anxiety (P8).*

*People around me are saying a lot of things, but I am following what comes to my mind. I trust my doctor. I ask my questions to him, and I follow his suggestions (P3).*

*My biggest supporter in this process is my husband. When I have a problem, I immediately call him. He immediately finds a solution or consoles me when I weep (P10).*

#### Physical

*There are many changes happening in my body. I have nausea, I feel tired, I have pain, I can’t lift my arms, I am gaining weight, and I can’t breathe. But these are temporary things that will disappear when this process is over, so I don’t care too much about them. They do not matter as long as my baby arrives healthy (P8).*

*Gaining weight makes me unhappy. I feel ugly. I want to give birth as soon as possible. These changes affect me very much. There are times when I cannot keep up with normal life (P10).*

*I haven’t experienced much change because it’s not my first pregnancy. I know the changes and I’m used to them. I’m very grateful for my situation (P11).*

## Faith and Spirituality During Pregnancy

### Establishing a Relationship with God

*I didn’t want it. I even thought about having an abortion. God gave it to me as a surprise. I am expecting a baby girl. I feel closer to God. How rebellious a human being I have been and how generous God is! (P5).*

*In my opinion, this is the greatest privilege that God, the Creator, has given to women. To be able to give birth to a living being! It is not something we do. God bestows it upon us (P9).*

### Reading the Holy Quran

*I’m preparing my baby from today. It seems more attractive to me that it grows up listening to the verses of the Holy Quran spiritually, rather than listening to the sounds of television and other things (P13).*

*Normally, I read the Holy Quran. I am going to read it all. You especially read Surahs Yasin and Tebareke a lot, but since I got pregnant, I have especially started reading Surah Yusuf and Surah Maryam, and I want those around me to read these Surahs to me (P3).*

### Performing the Daily Five Time Prayers

*I have already been performing my daily prayers. Now I have added my baby to my prayers. I am praying to God so that I will have a healthy child, God will bestow it upon me, it will have a good future, and it will be a good child(P4).*

## Support for the Moment of Birth

### Praying

*God forbid, if something happens to me, what will happen to my baby? I pray a lot for both of us to get out of this process healthily (P16).*

*I believe that there are some Surahs to be read or glorifications of God for a good birth; I turn to them. I believe they will give me strength. I think it represses my concerns. I’m trying to calm down a little more with them* (*P13).*

## Motherhood

### Dedication

*Thinking about an individual before yourself means being able to become “us” not “me”. Until you give birth, your priority is always “me”. But then priorities change. Your baby becomes the priority in every aspect, emotionally, materially, and spiritually, in terms of needs and desires (P12).*

*You sacrifice your sleep before you even hold it in your arms. You sacrifice your desires. You want to eat something but you can’t for the sake of its health. You want to go somewhere, but you cannot go in case it gets hurt. Motherhood is definitely sacrifice and patience. (P3).*

### Charge

*A child is a charge bestowed upon you. You have to prepare it for the world. (P14).*

*There is a living being that is dependent on you and has been entrusted to you. You try to raise it properly, faithfully, and sincerely(P4).*

#### Responsibility

*It needs you and you need to meet its every need. In my opinion, a person who cannot afford these should not be a mother. For this reason, the mother should be knowledgeable and educated. (P2).*

*I think there is no perfect mother. Of course, every mother has mistakes. I will pay attention to these too. I will try to fulfill all my responsibilities as much as I can (P15).*

*It is important not only to take care of the child physically, of course, to feed, wash, and raise it, but it is also necessary to feed the child spiritually, by loving, kissing, and embracing it (P11).*

*I am already thinking about my baby. My priorities have changed and I am already planning what I will do (P14).*

*At first, I was very scared, I thought a lot about whether I could take full responsibility for a baby, take care of it, and be sufficient. When I felt ready, I got pregnant and when my husband said he would take responsibility, I was very relieved. We decided it together (P5).*

*I used to not pay attention to myself, but now I pay more attention because there is a living creature inside me. I am now responsible for its healthy birth (P6).*

#### Protecting

*I am very careful about myself because my nutrition and sleep will affect my baby (P4).*

*I work and have a stressful job. As this stress may affect my baby, I am trying to stay away from it as much as possible. I am doing something to relax myself (P14).*

*I am not watching TV* *,* *especially the news, thinking it may be influenced by my mood. I want not to hear bad news from my circles or television (P15).*

#### Patience

*Raising a child is a very difficult and tiring process. I think God will give me patience. I understand my mother better now (P5).*

## Discussion

Spirituality about pregnancy and birth is one of the issues that should be addressed in this process. Religious practices, as a part of spirituality, have an important place in pregnancy (Adanikin et al., 2014; Ghodrati et al., 2018). Pregnant women use religious practices for divine healing as a tool to cope with the pregnancy-related symptoms they experience during this period (Crowther & Hall, 2015; Jesse et al., 2007).

In our study, pregnant women stated that they became pregnant to become mothers, start a family, and give meaning to life. During this period, women pay more attention to their own health to have a problem-free pregnancy and give birth to a healthy baby (Naser, 2012). Although the problems caused by the physical and psychological changes experienced during this period are tried to be alleviated with preventive health behaviors, such as nutrition, rest, exercise, and check-ups, women also value religion and other practices (Heidari et al., 2015; Liamputtong et al., 2005).

In addition, religion provides spiritual support to pregnant women outside of the medical process (Fouka et al., 2012). In our study, the pregnant women believed that the birth of a miraculous being such as a baby and the bestowal of this responsibility to women were a blessing and gift from God. In a study, a midwife witnessed a pregnant woman quitting drug abuse and starting to pray and stated that most pregnant women living in their area went to temples for worshipping (Ohaja et al., 2019). After doing everything they can, pregnant women seek support through prayers and want to meet their spiritual needs. The common belief is that prayers help communicate with the divine power and bring individuals closer to the Creator, and that help can only come from the Creator, as external support in this process (Crowther & Hall, 2015).

In our study, participants stated that they continued to pray for a painless and comfortable birth. In a similar study, pregnant women stated that they became closer to God at the time of birth and felt strong communication between them. They also stated that they asked God for help to relieve their pain and were thankful after the birth was completed (Callister & Khalaf, 2010). In a study by Bahar et al. (2005), pregnant women stated that they performed ablution and prayed before going to the hospital for birth (Bahar et al., 2005). On the other hand, in Nepal, there is a belief that the house is religiously sacred and therefore cannot be defiled by giving birth at home, which protects the health of the mother and the baby (Kaphle et al., 2013). Since the moment of birth is the final point of this difficult process, it can be said that pregnant women pray more intensely during this time.

Religious practices during pregnancy vary in each religion. The basis of the practices is for the relaxation of the pregnant woman and avoiding complications. In our study, it was observed that pregnant Muslim women tended to read special Surahs and the Quran, perform daily prayers, pray, and recite the Tasbeeh. There are also practices performed by pregnant women and priests in Christianity. Pregnant women perform practices, such as praying, singing hymns, attending thanksgiving in church, and using religious objects such as anointing oil, blessed water, blessed white handkerchief, and holy sand, while priests say prayers and recite revelations for pregnant women, interpret bad dreams into good ones, stretch out hands, and anoint women (Aziato & Omenyo, 2016).

Historically, birth and motherhood have been seen as situations that develop spirituality (Callister & Khalaf, 2010). In our study, it was determined that pregnant women were aware that the baby was a gift given by God and that they knew their role in caring for it. They emphasized concepts, such as sacrifice, responsibility, protection, charge, and patience.

## Strengths and Limitations

The most important strength of the research is that it is the first study in Türkiye that allowed pregnant women to express their thoughts about the relationship between pregnancy and religion and spirituality. The limitations of the research are that the result cannot be generalized to the whole population since it has a qualitative study design and the small cohort.

## Conclusion and Recommendations

Religion, an important component of spirituality, can be effective in protecting the holistic health of pregnant women, who want to feel safe and protected by a divine power during this process. Nurses can help women go through the process more easily by supporting and guiding them in spiritual practices by evaluating the anxiety and fear they experience during this process. At this point, spirituality-based practices can be included in nursing education.

## Declarations

### Confict of interest

The authors declare no potential conficts of interest with respect to the research, authorship, and/or publication of this article.
